# Alleged Infective Dangers of the Communion Cup

**Published:** 1911-08-19

**Authors:** 


### Alleged Infective Dangers of the Communion Cup.

Once more the possible hygienic dangers of the communion cup are being discussed in the daily papers. Observations made at the recent conference on tuberculosis by Dr. Marcus Paterson seem to have been responsible for this renewal of discussion on what most people regard as a difficult and delicate subject. Fortunately for us, delicacy is a lay, not a scientific, bogey for the restriction of discussion. Defence of the method of administration employed in the Church is impossible, hygienically speaking, but does there exist any authoritative evidence of actual danger? This is the question an answer to which has been reasonably demanded by celebrants.

The physical possibility of the transmission of disease is beyond question in the circumstances, even if the cup be wiped after having touched the lip of each communicant—a method practised, but often perfunctorily, in a few churches. But it may well be asked if the danger is not generally greatly exaggerated in the case of every participator except the celebrant himself. In those churches where the washing of the vessels in the presence of the communicants, which is enforced by the rubric, is carried out to a meticulous extreme, the doctor cannot refrain from pointing out that the celebrant sterilises the cup at the expense of his own alimentary tract. Samuel Butler, a scientist and, according to his own words, a serious Churchman, spent his life in enforcing the lesson that pure logic pursued for its own sake ends in ridiculous extremes, and that conscientious Laodiceanism can be made a most practical guide to conduct. This is the point we wish respectfully to drive home; let the spirit of the rubric be preserved, but not at the expense of a letter which may prove deadly to individual lives.

We now have to deal with the possibility of risk to the communicants. In the first place, it cannot be emphasised too strongly that it is quite impossible at present to obtain reliable statistics on the incidence, say, of consumption among communicants and non-communicants, of communicants after the Roman Catholic rite, or indeed of communicants at all. The clergy of all religious bodies have, like the laity, shunned statistical methods on any subject; let us remember that the weather reports and the very census itself are only half a century old. Modern science depends on the growing practice of obtaining statistical information on intelligent and instructed lines. Until this be done, it is unlikely that any cases of tuberculosis can be traced to the communion cup, especially when we bear in mind that the opportunities of ingesting tubercle bacilli in the daily round are so many, and that even regular attendance at the communion-table is, annually considered, so comparatively rare an event. The chalice itself presents usually a highly polished or plated surface, and the wine has probably some antiseptic properties. This, however, is not enough; there is one practical scientific method now in use which we are somewhat chary of mentioning, because it is at present chiefly, if not exclusively, practised by one section of Christendom. We hope, however, that this fact will not damn it in the eyes of all the rest. The practice in question, which we applaud from its hygienic value, is that of certain branches of the Free Churches, notably the Baptists, and consists in the substitution of a number of small cups in place of the one or two usually to be found in use in the Established Church. Of course, those sectarians who are anxious to stir up strife will say that it is impossible to have a sufficient number of these cups with which to serve each individual communicant separately Our answer is that this should be no more difficult than it is found to be in the hourly communions of the great catering establishments. What organisation can do and has done for a purely secular purpose, organisation can and should do for the performance of a sacred rite.

The real danger lies in the terrible ease with which such a transmissible disease as syphilis can be passed on by means of, say, a labial infection, the nature of which may have been unsuspected by the infecting communicant. We can hardly believe that any knowingly infected person would be so criminally cynical as to attend a ceremony of this character, though it is stupidity, which is another name for want of imagination, which is generally enough to account for actions of the kind. We have no desire to be alarmist over this matter; and we firmly believe that there is no cup in the world that presents a danger approximating in the least degree to that of the metal cups hung on chains at our public fountains.

